# Mechanisms of Acupuncture Therapy in Ischemic Stroke Rehabilitation: A Literature Review of Basic Studies

**DOI:** 10.3390/ijms18112270

**Published:** 2017-10-28

**Authors:** Lina M. Chavez, Shiang-Suo Huang, Iona MacDonald, Jaung-Geng Lin, Yu-Chen Lee, Yi-Hung Chen

**Affiliations:** 1 Graduate Institute of Acupuncture Science, China Medical University, Taichung 40402, Taiwan; linita_salamanca@hotmail.com (L.M.C.); iona2005@mail.cmu.edu.tw (I.M.); 2 Department of Pharmacology and Institute of Medicine, College of Medicine, Chung Shan Medical University, Taichung 40402, Taiwan; sshuang@csmu.edu.tw; 3 School of Chinese Medicine, China Medical University, Taichung 40402, Taiwan; jglin@mail.cmu.edu.tw; 4 Department of Acupuncture, China Medical University Hospital, Taichung 40402, Taiwan; 5 Research Center for Chinese Medicine and Acupuncture, China Medical University, Taichung 40402, Taiwan; 6 Department of Photonics and Communication Engineering, Asia University, Taichung 41354, Taiwan

**Keywords:** acupuncture, cerebral ischemia, basic research, stroke, rehabilitation

## Abstract

Acupuncture is recommended by the World Health Organization (WHO) as an alternative and complementary strategy for stroke treatment and for improving stroke care. Clinical trial and meta-analysis findings have demonstrated the efficacy of acupuncture in improving balance function, reducing spasticity, and increasing muscle strength and general well-being post-stroke. The mechanisms underlying the beneficial effects of acupuncture in stroke rehabilitation remain unclear. The aim of this study was to conduct a literature review, summarize the current known mechanisms in ischemic stroke rehabilitation through acupuncture and electroacupuncture (EA) therapy, and to detail the frequently used acupoints implicated in these effects. The evidence in this review indicates that five major different mechanisms are involved in the beneficial effects of acupuncture/EA on ischemic stroke rehabilitation: (1) Promotion of neurogenesis and cell proliferation in the central nervous system (CNS); (2) Regulation of cerebral blood flow in the ischemic area; (3) Anti-apoptosis in the ischemic area; (4) Regulation of neurochemicals; and, (5) Improvement of impaired long-term potentiation (LTP) and memory after stroke. The most frequently used acupoints in basic studies include Baihui (GV20), Zusanli (ST36), Quchi (LI11), Shuigou (GV26), Dazhui (GV14), and Hegu (LI4). Our findings show that acupuncture exerts a beneficial effect on ischemic stroke through modulation of different mechanisms originating in the CNS.

## 1. Introduction

Stroke is defined as an acute focal injury of the central nervous system (CNS) arising from a vascular cause such as cerebral infarction, intracerebral hemorrhage, or subarachnoid hemorrhage, as described in a recent text; “rapidly developing clinical signs of focal (or global) disturbance of cerebral function, lasting more than 24 h or leading to death, with no apparent cause other than that of vascular origin” [1]. In 2013, stroke was the second-leading global cause of death after ischemic heart disease, representing 11.8% of total deaths worldwide [2]. The worldwide burden of ischemic and hemorrhagic stroke increased significantly between 1990 and 2010 [3]. Of the two major classes of stroke, ischemic (~80% of strokes) and hemorrhagic (~20% of strokes), this review concentrates on the ischemic type.

### 1.1. Pathophysiology of Ischemic Stroke

The ischemic cascade is a heterogeneous phenomenon, in which one event can cause or be caused by many other events [4]. The primary event in 85–90% of acute strokes is compromised vascular supply to the brain, which is highly vulnerable to ischemic insult, due to its low respiratory reserve and dependence on aerobic metabolism. The extent of damage depends on the duration, severity, and location of ischemia [5]. Macroscopic changes in the brain tissue and computed tomography (CT) or magnetic resonance imaging (MRI) changes through the course of a stroke serve as a guide to classify the evolution time from the beginning of the symptoms into three stages [6]: (1) Acute (up to 48 h); (2) Subacute (48 h to weeks); and (3) Chronic (weeks to months). Within minutes to hours, local depletion of oxygen and glucose will cause: (1) disruption of the ion gradient, resulting in cytotoxic edema and release of excitatory neurotransmitters (e.g., glutamate from the astrocytes) [5]; and (2) a switch from aerobic metabolism to anerobic metabolism, leading to metabolic acidosis [7]. These primary events lead to acute necrosis or cell death [4]. During the subacute stage of stroke, an upregulation of early genes and stress signals drive apoptosis and activation of the inflammatory cascade. At the same time, neuroprotective mechanisms are triggered by Akt pathways and neurotrophic factors [5]. The chronic stage involves processes for recovery and repair, in which neurogenesis, angiogenesis, and synaptogenesis predominate [8]. 

### 1.2. Treatment of Ischemic Stroke in Western Medicine

Following focal cerebral ischemia, the primary treatment goal is to prevent or reverse brain injury and to optimize cerebral perfusion in the surrounding ischemic penumbra by dilating blood vessels. Anticoagulant and thrombolytic therapy can promote the recovery of nervous function [9].

Administration of recombinant tissue plasminogen activator (rtPA) within 3 h of stroke onset improves clinical outcomes; large vessel occlusions that fail to open with intravenous (IV) rtPA alone are candidates for endovascular revascularization, by direct intra-arterial administration of a thrombolytic agent or by endovascular mechanical thrombectomy [9]. Antithrombotic treatment includes antiplatelet use of aspirin within 48 h of stroke onset, which reduces the risk of recurrence and mortality [9].

### 1.3. Associated Impairments and Rehabilitation

The most common impairments due to stroke include motor and sensory loss or alteration [10]. Rehabilitation stages involve medication, early physical, occupational, and speech therapy, all of which are intended to assist with the patient’s fast recovery and adaptation to daily life activities [9,11].

Other rehabilitation therapies include exercise programs to improve aerobic fitness and/or muscle strength, restriction of the non-paretic upper limb, repetitive task training for the paretic arm, bilateral arm training, gait and treadmill retraining, walking aids, intramuscular botulinum toxin administration for spasm, splinting to prevent and treat contractures, neuromuscular electrical stimulation, and acupuncture [10].

### 1.4. Acupuncture and Ischemic Stroke

Acupuncture is one of the oldest and most studied techniques in Chinese medicine, a procedure involving insertion of a fine needle into the skin or deeper tissues at specific locations (acupoints) of the body. This needling may be manipulated manually, electrically, or by heat [12]. Recent research suggests that acupoints may be excitable muscle/skin-nerve complexes containing a high density of nerve endings [13]. Manual acupuncture or electroacupuncture (EA) at particular acupoints activates afferent fibers that send signals to the spinal cord [14]. In the CNS, endogenous opioids are the principal biological mediators of the therapeutic actions of this ancient technique. Recently, several classes of molecules, such as neurotransmitters (catecholamine, acetylcholine, serotonin, glutamate, and γ-aminobutyric acid [GABA]), neuropeptides, cytokines, and growth factors have been identified as possible mediators for specific acupuncture effects [15]. Functional MRI (fMRI) and diffusion tension imaging (DTI) have been used to examine the neuronal specificity of acupoints [16,17].

Clinical trials have shown that acupuncture can enhance balance [18], reduce spasticity [19], and increase muscle strength [20]. A systematic review and meta-analysis of randomized trials conducted in 2010 has indicated that acupuncture may be effective in improving post-stroke impairment, as measured by its analgesic effect, motor rehabilitation, increased perfusion within peri-infarcts and low perfusion zones in the affected lobe, and the stimulation of neuronal reorganization, amongst other findings [21]. However, the poor study quality and publication bias hinder the strength of this recommendation. The reviewers call for large, transparent, and well-conducted randomized clinical trials that can help to implement the use of acupuncture in clinical practice [21]. The exact mechanisms underlying the beneficial effects of acupuncture in the treatment of stroke remain unclear.

This review of the literature examined what is known about the mechanisms involved in the efficacy of acupuncture for ischemic stroke rehabilitation, reducing post-stroke infarct volume and neurological deficit. According to the preclinical evidence in this review, the benefits of ischemic stroke rehabilitation are achieved through five different major mechanisms: (1) promotion of proliferation of CNS-resident cells; (2) regulation of cerebral blood flow via angiogenesis and modulation of vasoactive mediators; (3) anti-apoptosis via direct intervention in the intrinsic and extrinsic pathways or related pathways; (4) regulation of neurochemicals involved in the ischemic cascade; and (5) potentiation and recovery of hippocampal memory and learning processes.

## 2. Methods

We designed our literature review to include basic studies that addressed the mechanisms underlying the effects of acupuncture treatment in models of post-ischemic stroke injury. The literature search was performed in PubMed, restricted to English-language, full-text studies published from 1 January 2000 up until 31 December 2015, using the following keywords:

- Acupuncture
- Cerebral ischemia
- Effect

Inclusion criteria:

- Specific mechanisms describing how acupuncture exerted its effects
- Ischemic stroke type
- Exclusive use of acupuncture after the ischemic injury
- Related studies cited in these articles

Using these parameters, we identified 105 published articles. From those, only 22 matched the inclusion criteria and were reviewed. The references of these 22 articles identified an additional 18 articles that also matched our inclusion criteria, so these were included in the final review, with 40 articles in total. All of the studies performed similar animal experimental models, involving permanent or temporary occlusion of the unilateral or bilateral middle cerebral artery, internal, external, or common carotid artery. In all studies, acupuncture or electroacupuncture was performed after the surgery.

## 3. Results

The 40 studies included in this review employed similar experimental models, using Sprague Dawley rats, Wistar rats, mice, or gerbils, with permanent or temporary occlusion of the unilateral or bilateral middle cerebral artery and/or common carotid artery. Acupuncture or EA was performed mostly in the acute and subacute stages after ischemic injury. These studies reveal five major categories classifying the principal mechanisms underlying the effects of acupuncture in stroke rehabilitation.

### 3.1. Promotion of Neurogenesis and Cell Proliferation in the Central Nervous System (CNS)

During ischemic stroke, the cell population in the CNS is affected and two major zones develop, according to the degree and duration of ischemia; the ischemic core, where immediate cell death occurs, and the ischemic penumbra, where despite the initial damage, the structure of the tissue is intact but the function is altered. Due to the reversible damage, this zone represents the most important target in acute stroke therapy [4]. Acupuncture promotes cell proliferation in the CNS after ischemic stroke via two different mechanisms; first, by neurogenesis, which is limited to neurogenic areas (the subventricular zone of the lateral ventricle [LV] and the dentate gyrus [DG] of the hippocampus) in adulthood. Second, acupuncture promotes cell proliferation in ischemic-affected tissue and some contiguous zones to the injury produced by the occlusion of the middle cerebral artery.

#### 3.1.1. Neurogenesis

Acupuncture improves the division of stem cells by enhancing GSK-3β/PP2A expression, increasing neurotrophic factors BDNF/VEGF (brain-derived neurotrophic factor/vascular endothelial growth factor), and upregulating neuroprotective substances such as retinoic acid.

GSK3β/PP2A is a group of proteins that relate to biological processes of phosphorylation and cell proliferation. GSK3β controls protein synthesis, cell proliferation, differentiation and apoptosis; PP2A downregulates GSK3β activity by dephosphorylation. This balance in the activity of both proteins and the regulation of their expression correlates with the control of CNS neurons, enhances neurogenesis at the hippocampus, and improves cerebral blood flow in the ischemic cortex, hippocampus, and striatum. BDNF and VEGF serve as mediators for survival cascades of neural stem cells; they stimulate adult neurogenesis, enhance the birth of new neurons and neuronal migration in neurogenic areas. VEGF alone constitutes a major angiogenic factor. EA increases the number of neuroblasts in the subventricular zone and hippocampus, and the expression of BDNF and VEGF mediating PI3K activation in the same neurogenic areas [22]. EA stimulation also promotes the expression of retinoic acid, which is capable of enhancing neurogenesis in the subventricular zone and the hippocampus [23]. The study evidence in support of this section can be seen in the supplementary file (Table S1).

#### 3.1.2. Cell Proliferation in Ischemic Tissue

Acupuncture has been shown to promote cell proliferation in tissue damaged by ischemia. Affected areas involve the cortical peri-infarct area, ischemic and non-ischemic cortex, LV, and striatum. Following cerebral ischemia/reperfusion, EA treatment activates the Wnt/β-catenin pathway, which encourages the proliferation of reactive astrocytes and neural progenitor cells [24]. EA also upregulates positive cells and mRNA expression of stem cell factors, c-kit and metallopeptidase-9 (MMP-9) [25], stimulates cerebral cell proliferation via activation of the ERK1/2 pathways and cyclin expression [26,27], and exerts neuroprotection via proliferation of glial fibrillary acidic protein/vimentin/nestin-positive reactive astrocytes and by enhancing BDNF expression in the peri-infarct cortex and striatum [28]. The study evidence in support of this section can be seen in the supplementary file (Table S2).

## 4. Regulation of Cerebral Blood Flow

In the ischemic cascade, the loss of vascular structural integrity results from direct injury or cell death, blood-brain barrier alteration, and microvascular injury [5]. Among the specific endothelial mechanisms related to stroke, active angiogenesis is one of the neuroprotective mechanisms that is triggered by ischemia [29]; formation of new blood vessels begins within 12–24 h following stroke and can continue for at least 21 days afterwards. It is strongly mediated by VEGF and other angiogenic factors, including angiogenin-1, amongst others [5], helping to restore the oxygen/nutrient supply to the injured tissue and providing an environment that enables neuroprotective processes such as neurogenesis.

During ischemia, vasodilative mediators in the acute stages are key to reducing the infarct lesion and associated secondary damage. The angiotensin system is counter-regulated by vasoactive substances; an elevation in nitric oxide synthase activity includes the detrimental types I and II, while type III improves blood flow via vasodilatation and inhibition of platelet adhesion, with an added antioxidative and anti-inflammatory effect [4]; another mechanism seeks to maintain cerebral perfusion by reducing vascular resistance through metabolic factors, myogenic processes, and direct endothelial mechanisms.

In our review, acupuncture was associated with two different subcategories of action that involve the regulation of the cerebral blood flow. 

### 4.1. Angiogenesis

EA at Hegu (LI4) enhances the expression of angiogenic factors such as VEGF and angiogenin-1 in the peri-infarct cortex, LV, and DG, and simultaneously inhibits the production of the antiangiogenic factor endostatin [30]. The study evidence in support of this section can be seen in the supplementary file (Table S3).

### 4.2. Vasoactive Modulation

Multiple dynamic responses to stroke develop in brain vascular structures. Specifically, injury to the endothelium is related to the impaired release of nitric oxide and increased endothelin-1 production, leading to an increase of the vascular tone that is detrimental in the infarct area and enhances the ischemic injury [4].

The vasoactive modulatory effect of EA at Dazhui (GV14), Baihui (GV20), and Shuigou (GV26) acupoints increases perfusion on the affected side by releasing acetylcholine, leading to the release of nitric oxide [31]. 

EA can also decrease the expression of angiotensin II and its type 1 receptor (AT1R), and thereby increases cerebral blood flow and vasodilation [32]. The study evidence in support of this section can be seen in the supplementary file (Table S4). 

## 5. Anti-Apoptosis

Post-stroke, neuronal populations undergo apoptosis, a regulated process of cell death with minimal inflammatory reaction or genetic material release [4].

The apoptotic signaling cascade after cerebral ischemia develops via two apoptotic pathways (intrinsic/extrinsic) [33]. Inhibition of apoptosis can reduce ischemic injury and prevent activation of cell death [7]. 

Apoptosis is mediated by caspases, particularly caspases 1 and 3, and can be triggered by the release of cytochrome C from mitochondria, through activation of the apoptosome in the intrinsic pathway [4]. The extrinsic pathway exerts its effects via other related processes that include the release of oxygen free radicals, death receptors, damage to DNA, activation of proteases and failure of the ion pump [7]. Apoptosis represents one of the most common mechanisms studied in stroke rehabilitation.

### 5.1. Specific Apoptotic Pathway

Manual stimulation at Zusanli (ST36), Hegu (LI4), Tanzhong (CV17), Zhongwan (CV12), Qihai (CV6), and Xuehai (Sp10) acupoints suppresses pro-apoptotic factors c-Fos, caspase-3 and Bax, and upregulates the expression of the protective protein, Bcl-2 [34,35].

EA at Baihui (GV20), Shuigou (GV26), and Qihai (CV6) acupoints has been shown to enhance the levels of anti-apoptotic factors such as Akt, Bcl-2, Bcl-xL, and cIAP1/2, as well as inhibit apoptotic mediators DR5 and caspases 3, 8, and 9 [36,37]. The study evidence in support of this section can be seen in the supplementary file (Table S5).

### 5.2. Non-Specific Apoptotic Pathway

Related apoptosis signaling pathways can also be affected by acupuncture; the initial injury mechanism triggers these non-specific pathways and incudes the expression of apoptotic mediators [33].

Studies of EA stimulation at multiple acupoints including Zusanli (ST36), Hegu (LI4), Quchi (LI11), Dazhui (GV14), Fengfu (GV16), Baihui (GV20), Shenting (GV24), Renzhong (GV26), Chengjiang (CV24), Chize (LU5), and Sanyinjiao (SP6) reveal the upregulation of anti-apoptotic-related factors, such as the Bcl-2/Bax ratio [38,39], via the activation of the PI3K/Akt, ERK1/2 [40,41] and p38 MAPK/CREB pathways [42], the downregulation of the S100B [43] and NF-kB pathways [44], and a reduction in the expression of TRPM7 [45] and JAK2 [46]. The study evidence in support of this section can be seen in the supplementary file (Table S6).

## 6. Regulation of Neurochemicals

The ischemic stroke cascade involves a wide variety of important complex processes. An initial failure in energy induced by insufficient oxygen and glucose leads to cell injury and death resulting from various interdependent molecular pathways that include excitotoxicity, acidotoxicity, ionic imbalance, oxidative and nitrative stress, inflammation, and apoptosis [7].

### 6.1. Neurotransmitters and Receptors

Ischemic injury results in neurological deficits through the release of excitatory neurotransmitters, uncontrolled discharge of glutamate, ionic imbalance, and the production of metabolic products, such as oxygen-free radicals [5]. 

Acupuncture stimulation can increase the levels of dopamine in cortex and hippocampus and thus reduce the degree of cerebral atrophy and improve neuronal deficits [47]. In Sprague Dawley rats, a single acupoint stimulation at Baihui (GV20) increased the dopamine levels in the cerebral cortex and hippocampus on the affected side and modulated neuronal plasticity by reducing the extent of cerebral atrophy after ischemia. Interestingly, the study researchers reported that acupuncture did not improve cognitive and memory impairments, despite an observed increase in dopamine levels [47].

One study that applied EA at the Shuigou (GV26) and Chengjiang (CV24) acupoints found that it reduced the overexpression of the neurotrophic factor NMDA NR1 in the ischemic cortex and prevented neuronal apoptosis [45].

Applying EA simultaneously to the Shendao (GV11) and Fengfu (GV16) acupoints depressed glutamate release in the CA1 hippocampal subfield, prevented excitatory-related injury, and suppressed hyperemia during reperfusion [48]. 

Application of EA to the Baihui (GV20) and Shuigou (GV26) acupoints increased GABA levels in the cerebral cortex and hippocampus CA1 regions, protecting them from excitotoxic damage and preventing ischemic injury [49]. EA also increased the percentage of surviving neurons in the cortex and striatum. In another paper that focused on GABA receptors, stimulation of an extra acupoint, Jiaji (EX-B2), increased the expression of GABA receptors, GABAAγ2 and GABABR2, in the striatum and spinal cord, as well as β-endorphin levels, and reduced infarct volume [50]. The study evidence in support of this section can be seen in the supplementary file (Table S7).

### 6.2. Antioxidant Enzymes

A major pathophysiological mechanism related to ischemic injury concerns oxidative and nitrative stress. The high metabolic activity of the brain relies on constant oxygen and glucose supply from the circulation. Extremely low storage levels of energy and metabolites within the brain mean that it is especially vulnerable to interruptions in blood flow [51]. The antioxidative effects of acupuncture can be seen by manual stimulation at the Tanzhong (REN17), Zhongwan (REN12), Qihai (REN6), Zusanli (ST36), Xuehai (Sp10), and Baihui (GV20) acupoints in cerebral multi-infarction rats, where acupuncture increases the activity of antioxidant enzymes superoxide dismutase (SOD) and glutathione peroxidase (GSH-Px) in the hippocampus [52], and inhibits NAPDH oxidase-mediated oxidative stress in two-vessel occlusion rats [53]. Electroacupuncture also results in an antioxidative effect through stimulation at the Fengchi (GB20), Renzhong (GV26), Baihui (GV20), and Zusanli (ST36) acupoints, resulting in increases in the levels of SOD and GSH-Px [54], succinic dehydrogenase, DADH dehydrogenase, cytochrome C oxidase [55], and the thioredoxin system [56], indicating that EA promotes neurological recovery by increasing the levels of antioxidative mediators. The study evidence in support of this section can be seen in the supplementary file (Table S8).

### 6.3. Inflammatory Mediators

The inflammatory reaction following cerebral ischemia is primarily mediated by cells from microglia, astrocytes, and leucocytes, as well as by different substances, including cytokines (interleukin-1 [IL-1], IL-6, and IL-10, and tumor necrosis factor-α [TNFα]); this inflammatory reaction induces gene expression and activation of transcription factors involved in the regulation of the ischemic cascade [4]. EA at the Zusanli (ST36), Baihui (GV20), and Quchi (LI11) acupoints exerts an anti-inflammatory effect by inhibiting the local release of cytokines including TNFα and inhibiting alarmin heat shock protein (HSP70) [57] and Toll-like receptor 4/nuclear factor-kB (TLR4/NF-kB) signaling [58]. The study evidence in support of this section can be seen in the supplementary file (Table S9).

### 6.4. Neurotrophic Factors

Neurotrophic factors are related to the proliferation and maturation of neurons and mediate neural regeneration in cerebral ischemia. BDNF is one of the most potent growth factors involved in recovery following stroke. EA treatment at the Baihui (GV20) and Qubin (GB7) acupoints increases the levels of BDNF at the ischemic lobe and improves functional and motor recovery [59]. Similarly, investigations into the promotion of cell proliferation in ischemic tissue and neurogenic areas [22,28] and non-specific apoptotic pathways [38] have also found that acupuncture enhances the expression of BDNF. The study evidence in support of this section can be seen in the supplementary file (Table S10).

### 6.5. Anerobic Metabolism

Following ischemia, the resultant low levels of glucose and oxygen lead to bioenergetic failure, forcing metabolic changes and anerobic glycolysis and lactate accumulation [4]. Elevated lactate levels are a hallmark of anerobic metabolism in stroke and are a probable cause of secondary damage [4]. Nevertheless, higher amounts of lactate production play a neuroprotective role in the recovery period after ischemia, serving as a major energy substrate generated by astrocytes for surviving neurons in the injured brain [60]. In the acute stage of stroke recovery, EA at the Neiguan (PC6) and Quchi (LI11) acupoints increases lactate concentration in the ischemic brain for utilization by injured neurons [60]. The study evidence in support of this section can be seen in the supplementary file (Table S11).

## 7. Acupuncture Modulates Long-Term Potentiation and Improves Memory

LTP is a cellular model of learning and memory formation of synaptic transmission in the hippocampus [61]. In ischemic stroke animal models, the CA1 hippocampal subfield is impaired and acupuncture improves this process by modulating different pathways, upregulating cAMP/PKA/CREB [61] and decreasing the expression of NR1-TRPV1 [62]. 

Manual acupuncture at Zusanli (ST36) and EA at Baihui (GV20) improves cognitive hippocampus function by modulating cAMP/PKA/CREB [61] and by reducing the expression of NR1-TRPV1 [62], thus reducing deficits related to LTP due to excitotoxicity. The study evidence in support of this section can be seen in the supplementary file (Table S12).

## 8. Summary of Main Acupoints Selected in the Reviewed Studies

Among the 40 articles included in this review, the six most frequently used acupoints were (Table 1): (1) Baihui (GV20), (2) Zusanli (ST36), (3) Quchi (LI11), (4) Shuigou (GV26), (5) Dazhui (GV14), and (6) Hegu (LI4) (Table 1).

Baihui: Baihui is implicated in the modulation of neurotransmission by increasing dopamine levels [47] and improving memory via LTP recovery in the hippocampus [62]. Zusanli: improves cognitive hippocampus function by modulating the cAMP/PKA/CREB signaling pathway [61], stimulates neurogenesis [23,24,26,27,28], exerts a neuroprotective role against oxidative damage [34,35,38,39,52,53,56], has an anti-apoptotic effect [41] and improves the expression of different anti-inflammatory mediators [57,58]. Quchi: promotes neuronal cell proliferation [23,24,26,27,28], activates PI3K/Akt pathways to prevent apoptosis [38,39], reduces ischemic brain damage through anti-inflammation [58] and improves metabolism in the affected area [60]. Shuigou: one of the most commonly used single acupoints, induces neurogenesis by stimulating the GSK3β/PP2A signaling pathway [63] and modulates the angiotensin system by reducing the expression of angiotensin II and AT1R, while also enhancing the expression of AT2R, which is related to angiogenic processes [32]. Dazhui: enhances the proliferation of neuronal stem cells [22], improves perfusion at the ischemic zone [31], and mediates anti-apoptotic pathways [40,43,46]. Hegu: this acupoint is involved in cell proliferation in ischemic tissue and angiogenesis: first, by increasing stem cell factor expression in peri-infarct tissues [25]; second, by upregulating angiogenic factors such as VEGF and angiogenin-1; and third, by suppressing endostatin expression [30].

The most common combinations of acupoints were as follows:

Zusanli + Quchi: this combination involved the following mechanisms: Neurogenesis: increased production of retinoic acid [23]. Cell proliferation in ischemic tissue: activation of the Wnt/β-catenin pathways [24], ERK1/2 pathways [26,27], and increased expression of cell cycle proteins and BDNF [28]. Anti-apoptosis: upregulation of the PI3K/Akt pathway [38,39]. Anti-inflammatory activity: suppression of the TLR4/NF-kB pathway [58]. Dazhui + Baihui: this was the second most used acupoint formula and involved the following mechanisms: Neurogenesis: increased levels of neurotrophic factors including BDNF and VEGF [22]. Release of vasodilative mediators: increased acetylcholine and endothelial nitric oxide synthase discharge at the ischemic cerebral cortex [31]. Anti-apoptosis: activated the MEK1/2/ERK1/2/p90RSK/bad signaling pathway [43] and suppressed JAK2-mediated apoptosis [46]. Baihui + Shuigou: together, these acupoints exert an anti-apoptotic effect by activating Akt-mediated pathways and suppressing pro-apoptotic caspase-9 [37]. This combination also produces an antioxidative effect, enhancing the expression of respiratory chain-related enzymes such as succinic dehydrogenase, NADH dehydrogenase and cytochrome C oxidase in the penumbra zone [55]. Zusanli + Baihui: the stimulation of these acupoints results in an antioxidative effect, suppressing the activity of NADPH oxidase and its subunits in the hippocampus [53], and results in anti-inflammatory activities through inactivation of mediators, such as HSP70 and TNFα [57]. Shigou + Chengjiang: these produce anti-apoptotic effects by inactivating TRPM7 [45] and modulating neurotransmission, reducing the expression of the NMDA receptor NR1 in ischemic cortical areas [64].

## 9. Discussion and Conclusions

**Scheme 1:**
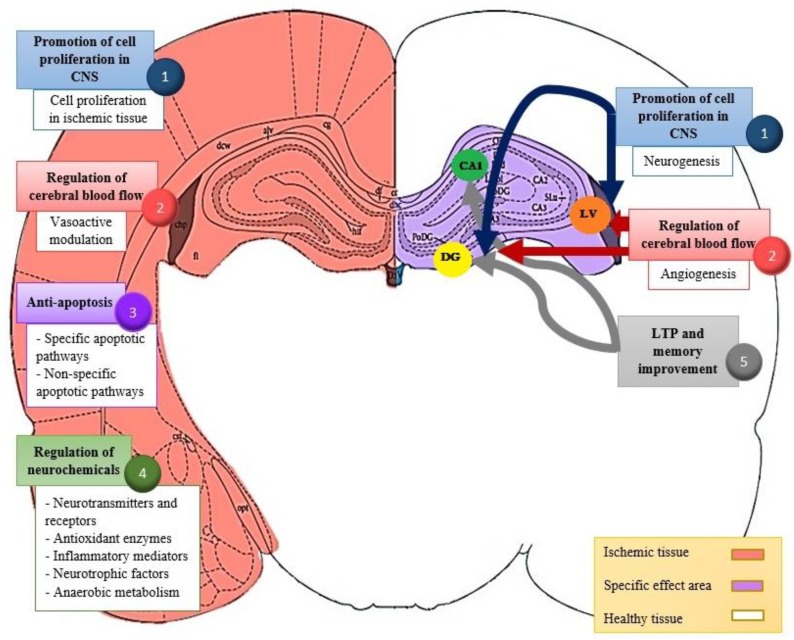
Acupuncture mechanisms in stroke rehabilitation.

In this review, acupuncture showed a beneficial effect on ischemic stroke rehabilitation in animal studies, through five different major mechanisms (Scheme 1): (1) promotion of cell proliferation in the CNS, limited to neurogenic areas and some ischemic tissues; (2) regulation of cerebral blood flow via angiogenesis and modulation of vasoactive mediators; (3) anti-apoptosis via direct intervention in the intrinsic and extrinsic pathways or related pathways; (4) regulation of neurochemicals involved in crucial steps in the ischemic cascade as neurotransmitters, antioxidants, inflammatory-related substances, neurotrophic factors, and metabolic substrates; and finally, (5) by potentiation and recovery of hippocampal memory and learning processes. Our findings are supported by those from two recently published literature reviews. The first, a systematic review and meta-analysis of preclinical studies published up to August 2015, assessed the effects of acupuncture treatment upon neurogenesis after experimental ischemic stroke [65]. It concludes that acupuncture appears to ameliorate neurological deficits and reduce brain edema in ischemic stroke, and that the mechanisms correlate positively with the enhancement of endogenous neurogenesis. The second paper, a Cochrane review of the efficacy and safety of acupuncture therapy in subacute or chronic ischemic or hemorrhagic stroke, concludes that acupuncture may improve global deficiency and some specific neurological impairments, with no obvious serious adverse sequelae, although most included trials were of limited quality and size [66]. Still further support is provided by the results and conclusions of four preclinical studies published in 2016, all of which demonstrate that EA improves neurological function after cerebral ischemia/reperfusion injury in rats [67,68,69,70].

The five major mechanisms involved in the beneficial effects of acupuncture/EA therapy in ischemic stroke rehabilitation are illustrated in Scheme 1. These five mechanisms are supported by evidence from the literature review, as follows: (1) Promotion of neurogenesis and cell proliferation in the central nervous system (showing neurogenesis in the subventricular zone of the lateral ventricle (LV) and the dentate gyrus (DG) areas in the hippocampus, and cell proliferation in ischemic tissue); (2) Regulation of cerebral blood flow in the ischemic area (showing angiogenesis in the LV and DG, and vasoactive modulation in ischemic tissue); (3) Anti-apoptosis in the ischemic area (through modulation of specific and non-specific apoptotic pathways); (4) Regulation of neurochemicals, such as: (a) Neurotransmitters and receptors, (b) Antioxidant enzymes, (c) Inflammatory mediators, (d) Neurotrophic factors, (e) Anerobic metabolism; and, (5) Improvement of impaired LTP and memory after stroke, via LTP enhancement in the DG and CA1 regions of the hippocampus. The arrows in the Scheme indicate the probable areas in the brain that relate to particular mechanisms.

There are acknowledged limitations associated with this type of literature review, which are explained as follows: (1) Under experimental conditions, ischemic stroke induced by occlusion of an artery can differ in diverse ways from a non-experimentally-induced stroke, such as the affected area and infarct volume, reperfusion time, etc. Thus, the evidence of benefits in preclinical studies of stroke cannot be completely extrapolated to the clinical environment. More clinical data are needed, to determine whether acupuncture is a realistic option. (2) Anesthesia is commonly used during acupuncture treatment in animals. We need to determine whether this practice alters the mechanisms of acupuncture. (3) The precise mechanism through which peripheral stimulation is transmitted from the acupoints to the brain and produces those neuroprotective effects as have been discussed in this manuscript remains unclear. (4) A literature review of this nature is limited by having to rely upon previously published research available on PubMed and the availability of these studies using the method as outlined and the appropriateness of these studies against the inclusion/exclusion criteria.

In general, acupuncture during the acute stages of stroke recovery reduces infarct volume and neurological deficits. We hope that future clinical trials will not only underline these findings, but also provide encouragement and hope to those millions of patients who experience the debilitating aspects of stroke.

## Figures and Tables

**Table 1:** Summary of main acupoints selected in the reviewed studies.

Acupoint	Frequency of Appearance
Baihui (GV20)	16
Zusanli (ST36)	16
Quchi (LI11)	9
Shuigou (GV26)	7
Dazhui (GV14)	5
Hegu (LI4)	4
Qihai (CV6)	3
Tanzhong (CV17)	2
Zhongwan (CV12)	2
Xuehai (SP10)	2
Fengfu (GV16)	2
Chengjiang (CV24)	2
Fengchi (GB20)	2
Shenting (GV24)	1
Chize (LU5)	1
Sanyinjiao (SP6)	1
Jiaji (Ex-B2)	1
Shendao (GV11)	1
Qubin (GB7)	1
Neiguan (PC6)	1

## Supplementary Materials

Supplementary materials can be found at www.mdpi.com/1422-0067/18/11/2270/s1.
