# Supplementary material for: Mechanisms of Acupuncture Therapy in Ischemic Stroke Rehabilitation: A Literature Review of Basic Studies
Source: Int J Mol Sci. 2017 Oct 28;18(11):2270. doi: 10.3390/ijms18112270 (PMC5713240; doi:10.3390/ijms18112270)
Supplement: Supplementary file 1 [file ijms-18-02270-s001.pdf]

Category I: Promotion of cell proliferation in CNS

Table S1: Neurogenesis

Study title	Authors	Publication	Target (receptor/ molecule/ pathway)	Ac type	Acupoints	Selection reason	Experimental model	Animal	Treatment	Stage	# sessions	Results	Conclusions
A study on the effect of neurogenesis and regulation of GSK3B/PP2A expression in acupuncture treatment of neural functional damage caused by focal ischemia in MCAO rats	Luo, D	Hindawi Publishing Corporation. Evidence-Based Complementary and Alternative Medicine. Vol. 2014, Article ID 962343	GSK3β/ PP2A (+)	MA	Shuigou (GV26)	Previous studies	Permanent Left MCAO	SD rats	Immediately after MCAO, **Duration:** Once daily for 3 consecutive days; **Stimulation:** Thrust/Lifted 3 times per/sec. for 1 min.	Acute	3	**1.** Ac. Improved CBF, **2.** Enhanced neurogenesis by upregulating the expression of GSK 3β and PP2A in cortex, hippocampus and striatum	Ac enhances neurogenesis via regulation of GSK-3 and PP2A expression
Electroacupuncture promotes post-stroke functional recovery via enhancing endogenous neurogenesis in mouse focal cerebral ischemia	Kim, Y	Plos One February 2014 Volume 9 Issue 2 e90000	BDNF/ VEGF (+)	EA	Dazhui (GV14), Baihui (GV20)	Not specified	MCAO for 40 min	Mice	5 days after MCAO; **Freq:** 2HZ; **Int:** 2V; **Time:** 20min; **Duration:** 10 consecutive days	Sub-acute	10	**1.** EA increased neuroblasts in the ipsilateral SVZ and hippocampus (Subcallosal zone) and enhanced differentiation into neurons or astrocytes, **2.** EA increased activation of BDNF and VEGF-mediated downstream PI3K at SVZ and hippocampus, **3.** EA improved division of NSCs	EA enhances proliferation and differentiation of neuronal stem cells via the BDNF and VEGF signaling pathway

Electroacupuncture promotes neurological functional recovery via the retinoic acid signaling pathway in rats following cerebral ischemia-reperfusion injury	Hong, J	International Journal of Molecular Medicine 31: 225-231, 2013.	Retinoic Acid (+)	EA	Zusanli (ST36), Quchi (LI11)	Previous studies	Left MCAO for 120 min	SD rats	**Freq:** 5/20Hz, **Int:** 2-4mA, **Time:** 20min, **Duration:** once a day for 4 weeks	Not specified	28	**1.** EA decreased infarct volume, **2.** promoted neurological functional recovery, and **3.** increased RA mRNA and protein expression	EA promotes neurological functional recovery through modulating RA expression and stimulation of neurogenesis

Table S2: Cell proliferation in ischemic tissue

Study title	Authors	Publication	Target (receptor/ molecule/ pathway)	Ac type	Acupoints	Selection reason	Experimental model	Animal	Treatment	Stage	# sessions	Results	Conclusions
Electro-acupuncture exerts beneficial effects against cerebral ischemia and promotes the proliferation of neural progenitor cells in the cortical peri-infarct area through the Wnt/β-catenin signaling pathway	Chen, B	International Journal of Molecular Medicine 36: 1215-1222, 2015	Wnt/β catenin (+)	EA	Zusanli (ST36), Quchi (LI11) on the paretic side	Previous studies	Left MCAO for 120 min	SD rats	1 day after MCAO; **Freq:** 1-20Hz; **Time:** 30min; **Duration:** once daily for 3 days	Acute	3	**1.** EA improved neurological deficits, **2.** reduced infarct volume, **3.** promoted proliferation of reactive astrocytes in peri-infarct cortex, **4.** enhanced proliferation of neural progenitor cells in peri-infarct cortex, and **5.** promoted activation of Wnt pathway in peri-infarct cortex	EA promotes the proliferation of neural stem/progenitor cells via the Wnt/β-catenin pathway
Electroacupuncture improves behavioral recovery and increases SCF/c-kit expression in a rat model of focal cerebral ischemia/reperfusion	Lu, T	Neurological Sciences (2013) 34:487–495	Stem Cell Factor (+)	EA	Hegu (LI4) bilateral	Not specified	Right MCAO for 120 min	SD rats	**Freq:** 40/60Hz; **Int:** 1mA; **Time:** 15 min; **Duration:** 7 consecutive days	Not specified	7	**1.** EA improved behavioral recovery, **2.** upregulated positive cells and mRNA expression of SCF, c-kit and MMP-9 after cerebral ischemia/reperfursion.	EA may promote neurobehavioral recovery by increasing the protein and mRNA expression of SCF, c-kit and MMP-9 after cerebral ischemia/reperfusion.

Electroacupuncture promotes neural cell proliferation in vivo through activation of the ERK1/2 signaling pathway	Huan g, J	International Journal of Molecular Medicine 33: 1547-1553, 2014	ERK1/2 (+)	EA	Zusanli (ST36), Quchi (LI11) on the paretic side	Not specified	Left MCAO for 120 min	SD rats	1 after MCAO; **Freq:** 1-20Hz; **Time:** 30min; **Duration:** once a day for 3 consecutive days	Acute	3	**1.** EA improved neurological function, **2.** reduced infarct volume, and **3.** activated the ERK1/2 signaling pathway in ischemic brain cortex.	EA appears to activate the ERK1/2 signaling pathway to protect against brain injury during cerebral ischemia
Electroacupuncture at Quchi and Zusanli treats cerebral ischemia-reperfusion injury through activation of ERK signaling	Xie, G	Experimental and Therapeutic Medicine 5: 1593-1597, 2013	ERK 1/2 (+)	EA	Zusanli (ST36), Quchi (LI11) on the paretic side	Clinical use	Left MCAO for 120 min	SD rats	2 h after reperfusion; **Freq:** 1-20Hz; **Time:** 30min; **Duration:** single	Acute	1	**1.** EA improved neurological function, **2.** reduced infarct volume, **3.** activated the ERK1/2 pathway in ischemic cerebral cortex and striatum, **4.** promoted cell proliferation, and **5.** EA enhanced expression of cyclin D1 and CDK4	EA enhances the activation of the ERK1/2 pathway and promotes cell proliferation
Electro-acupuncture at LI11 and ST36 acupoints exerts neuroprotective effects via reactive astrocyte proliferation after ischemia and reperfusion injury in rats	Tao, J	Brain Research Bulletin 120 (2016) 14–24	Cell cycle proteins and BDNF (+)	EA	Zusanli (ST36), Quchi (LI11) on paretic limb	Previous studies	Left MCAO for 120 min	SD rats	**Freq:** 1-20Hz; **Time:** 30min; **Duration:** 3 days	Not specified	3	**1.** EA improved neurological deficits, **2.** reduced infarct volume, **3.** improved motor function recovery, **4.** promoted reactive astrocyte proliferation in peri-infarct cortex and striatum, **5.** enhanced expression of cell cycle associated proteins (cyclin D1, CDK4, phospho Rb), and **6.** EA promoted secretion of BDNF from reactive astrocytes	EA promotes the proliferation of reactive astrocytes via enhancement of BDNF and promotes expression of cell cycle proteins

Category II: Regulation of CBF

Table S3: Angiogenesis

Study title	Authors	Publication	Target (receptor/ molecule/ pathway)	Ac type	Acupoints	Selection reason	Experimental model	Animal	Treatment	Stage	# sessions	Results	Conclusions
Effects of electroacupuncture on expressions of angiogenesis factors and anti-angiogenesis factors in brain of experimental cerebral ischemic rats after reperfusion	Ma, J	Journal of Traditional Chinese Medicine 2008; 28(3): 217-222	VEGF, Angiogenin 1 (+), Endostatin (-)	EA	Hegu (LI4) bilateral	Not specified	Right MCAO for 1h	Wistar rats	45 min after MCAO; **Freq:** 40-60Hz; **Int:** 1.5V; **Time:** 15min; **Duration:** once daily for 7 days	Acute	7	**1.** EA enhanced expression of VEGF and Ang-1 in cortex around the ischemic necrotic region, lateral ventricle and dentate gyrus of hippocampus, and **2.** EA reduced expression of endostatin	EA increases the expression of angiogenesis factors and inhibits the expression of anti-angiogenesis factors

Table S4: Vasoactive modulation

Study title	Authors	Publication	Target (receptor, molecule, pathway)	Ac type	Acupoints	Selection reason	Experimental model	Animal	Treatment	Stage	# sessions	Results	Conclusions
Electroacupuncture acutely improves cerebral blood flow and attenuates moderate ischemic injury via an endothelial mechanism in mice	Kim, J	Plos One February 2013 Volume 8 Issue 2 e56736	ACh/ Endothelial nitric oxide synthase (+)	EA	Dazhui (GV14), Baihui (GV20)	Not specified	MCAO for 60 and 90 min	Mice	Immediately after MCAO; **Int:** 1mA; **Freq:** 2Hz; **Time:** 20min; **Duration:** single	Acute	1	**1.** EA increased perfusion in the cerebral cortex **2.** increased ACh release and mAChR M3 expression in cerebral cortex, **3.** perfusion effects of EA were dependent on eNOS, **4.** EA decreased infarct volume after moderate ischemic injury	EA in the acute stage improves ACh/eNOS-mediated perfusion augmentation, improving tissue and functional recovery
Electroacupuncture improves cerebral blood flow and attenuates moderate ischemic injury via Angiotensin II its receptors-mediated mechanism in rats	Li, J	BMC Complementary and Alternative Medicine 2014, 14:441	Angiotensin II/AT1R (-), AT2R (+)	EA	Shuigou (GV26)	Previous studies	Right MCAO	Wistar rats	**Freq:** 15Hz; **Int:** 1mA, **Time:** 20min; **Duration:** single	Not specified	1	**1.** EA reduced neurological deficits, **2.** increased CBF at the ipsilateral and contralateral lesion sites, **3.** decreased expression of AngII, AT1R, and **4.** EA enhanced expression of AT2R in ischemic core and penumbra regions	EA significantly attenuates MCAO-induced increases in AngII expression and its receptor-mediated IP3 signal transduction pathway, reduces vasoconstriction and improves blood supply in ischemic region

Category III: Anti-apoptosis

Table S5: Specific apoptotic pathway

Study title	Authors	Publication	Target (receptor/ molecule/ pathway)	Ac type	Acupoints	Selection reason	Experimental model	Animal	Treatment	Stage	# sessions	Results	Conclusions
Acupuncture suppresses ischemia-induced increase in c-Fos expression and apoptosis in the hippocampal CA1 region in gerbils	Jang, M	Neuroscience Letters 347 (2003) 5-8	Caspase 3 (-)	MA	Zusanli (ST36) bilateral, Hegu (LI4) bilateral	Not specified	Common carotid artery occlusion for 5 min	Gerbils	Starting on the third day of the experiment; **Time:** 20min; **Duration:** Twice daily for 8 days	Sub-acute	16	**1.** Ac. suppressed ischemia-induced increases in c-Fos and caspase 3 TUNEL cells in the hippocampal CA1 region, **2.** The most potent inhibitory effect was observed at Zusanli acupoint	Ac. exerts anti-apoptotic effects in the hippocampal CA1 region by suppressing ischemia-induced increments in c-Fos and caspase 3 cells
Acupuncture protected cerebral multi-infarction rats from memory impairment by regulating the expression of apoptosis related genes Bcl-2 and Bax in hippocampus	Wang, T	Physiology & Behavior 96 (2009) 155–161	Bax (-) - Bcl-2 (+)	MA	Tanzhong (CV17), Zhongwan (CV12), Qihai (CV6), Zusanli (ST36), Xuehai (SP10)	Not specified	Right common carotid artery and external carotid artery occlusion	Wistar rats	Starting 16 days after artery occlusion; **Stimulation:** twisted twice a sec. for 30 sec. in each point; **Duration:** Once daily for 21 days	Chronic	21	**1.** Ac improved Morris water maze trial performance, **2.** Ac significantly reduced the number of apoptotic cells in hippocampal CA1 region, **3.** Ac upregulated Bcl-2 expression, and **4.** Ac counter-regulated the expression of pro-apoptotic Bax	Ac improves memory via anti-apoptotic effects in hippocampal CA1 region by downregulating the expression of pro-apoptotic factor Bax and upregulating the expression of anti-apoptotic factor Bcl-2

Potentiation of Akt and suppression of caspase-9 activations by electroacupuncture after transient middle cerebral artery occlusion in rats	Wang, S	Neuroscience Letters 331(2002) 115–118	Akt (+), caspase 9 (-)	EA	Baihui (GV20), Renzhong (GV26)	Not specified	Right MCAO for 90 min	Wistar rats	Just after MCAO; **Freq:** 20/3Hz; **Int:** 3mA; **Time:** 1h; **Duration:** single	Acute	1	**1.** EA increased expression of AKT mainly in the ischemic penumbra, **2.** EA diminished caspase 9 induction, and **3.** EA decreased number of TUNEL positive cells in the cortex	EA activates Akt and suppresses caspase 9 inhibiting apoptotic cascade
Effects of electroacupuncture on apoptotic pathways in a rat model of focal cerebral ischemia	Kim, Y	International Journal of Molecular Medicine 32: 1303-1310, 2013	DR5 extrinsic and intrinsic pathway mediators (-)	EA	Baihui (GV20), Qihai (CV6)	Previous studies	Right MCAO for 90 min	SD rats	Immediately after MCAO; **Freq:** 2Hz; **Int:** 1mA; **Time:** 30min; **Duration:** Twice a day	Acute	2	**1.** EA reduced infarct size, **2.** diminished the number of apoptotic cells in ischemic tissue, **3.** reversed the increase in expression of DR5, **4.** EA enhanced expression of Bcl-2, Bcl-xL, cIAP1 and 2, and **5.** EA decreased expression and activities of caspase-3, -8, and -9	EA neuroprotective effects may be associated with the inhibition of DR and mitochondrial apoptotic pathways

Table S6: Non-specific apoptotic pathway

Study title	Authors	Publication	Target (receptor/ molecule/ pathway)	Ac type	Acupoints	Selection reason	Experimental model	Animal	Treatment	Stage	# sessions	Results	Conclusions
Electroacupuncture at the Quchi and Zusanli acupoints exerts neuroprotective role in cerebral ischemia reperfusion injured rats via activation of the PI3K/Akt pathway	Chen, A	International Journal of Molecular Medicine 30: 791-796, 2012	PI3K/Akt (+)	EA	Zusanli (ST36), Quchi (LI11) on the non paretic limb	Clinical use	Left MCAO for 120 min	SD rats	120 min after MCAO; **Freq:** 1 and 20Hz; **Time:** 30min; **Duration:** single	Acute	1	**1.** EA improves neurological deficits, **2.** EA reduces infarct volume, **3.** EA activates the PI3K/Akt pathway, **4.** EA increases expression of BDNF and GDNF, and **5.** EA upregulates anti-apoptotic Bcl-2/Bax ratio in ischemic tissue	EA exerts neuroprotective function in ischemic stroke via activation of the PI3K/Akt anti-apoptotic pathway
Electroacupuncture at points of Zusanli and Quchi exerts anti-apoptotic effect through the modulation of PI3K/Akt signaling pathway	Xue, X	Neuroscience Letters 558 (2014) 14– 19	PI3K/Akt (+)	EA	Zusanli (ST36), Quchi (LI11) on the paretic limb	Clinical use	Left MCAO for 120 min	SD rats	1 day after MCAO; **Freq:** 4-20Hz; **Time:** 30min; **Duration:** once a day for 2 days	Acute	2	**1.** EA improved neurological deficit scores, **2.** reduced infarct volume, **3.** reduced apoptosis in penumbra of cerebral cortex, **4.** EA stimulated expression of PI3K and p-Akt in ischemic cortex, **5.** EA upregulated levels of p-Akt and Bcl-2, **6.** EA weakened the downregulation of p-Bad and Bcl-2, and reduced	EA exerts a neuroprotective effect on cerebral ischemia through the activation of the PI3K/Akt pathway, inhibiting apoptosis in the penumbra of cerebral ischemic cortex

												the upregulation of Bax, and **7.** EA enhanced the ratios of the protein of p-Bad/Bad and anti-apoptotic Bcl-2/Bax	
Electroacupuncture-like stimulation at Baihui and Dazhui acupoints exerts neuroprotective effects through activation of the brain-derived neurotrophic factor-mediated MEK1/2/ERK1/2/p90RSK/bad signaling pathway in mild transient focal cerebral ischemia in rats	Cheng, C	Complementary and Alternative Medicine 2014, 14:92	MEK1/2/ERK1/2/p90RSK/bad pathway (+)	EA	Baihui (GV20), Dazhui (GV14)	Chinese Medicine theory - Previous studies	Right MCAO for 15 min	SD rats	Following MCAO; **Freq:** 5Hz; **Int:** 2.7-3mA; **Time:** 25min; **Duration:** twice daily for 2 consecutive days	Acute	4	**1.** EA reduced infarct volume, **2.** EA lowered neurological deficit scores, **3.** EA enhanced BDNF expression in ischemic cortex, **4.** EA suppressed increase of caspase-3, and **5.** EA enhanced expression of protein kinases related to the ERK1/2 pathway	EA elicits BDNF-mediated neuroprotective action against caspase-3-dependent neuronal apoptosis through activation of the Raf-1/MEK1/2/ERK1/2 pathway
Electroacupuncture-like stimulation at the Baihui (GV20) and Dazhui (GV14) acupoints protects rats against subacute-phase cerebral ischemia-reperfusion injuries by reducing S100B-mediated neurotoxicity	Cheng, C	Plos One March 2014, Volume 9, Issue 3, e91426	S100B (-)	EA	Baihui (GV20), Dazhui (GV14)	Chinese Medicine theory - Previous studies	Right MCAO for 15 min	SD rats	After MCAO; **Freq:** 5Hz; **Int:** 2.7-3mA; **Time:** 25min; **Duration:** once daily for 6 consecutive days	Acute	6	**1.** EA reduced infarct volume, **2.** EA improved neurological deficits, **3.** EA lowered S100B, p38 MAP kinase-mediated NF-kB, and iNOS expression, **4.** EA downregulated TNF-α/TRADD/FADD/cleaved caspase-8/cleaved caspase-3 apoptotic pathway in ischemic cortical penumbra, **5.** EA	EA provides neuroprotection by downregulation of astrocytic S100B expression, delaying infarct expansion through the modulation of p38 MAP kinase-mediated NF-kB expression, attenuating oxidative/nitrative stress and downregulating the TNF-

											downregulated S100B/ nitrotyrosine	a/TRADD/FADD/ cleaved caspase-8/cleaved caspase-3 apoptotic pathway
Electroacupuncture at different frequencies (5Hz and 25Hz) ameliorates cerebral ischemia-reperfusion injury in rats: possible involvement of p38 MAPK-mediated anti-apoptotic signaling pathways	Cheng , C	Complementary and Alternative Medicine 2015 15:241	p38 MAPK/ CREB (+)	EA	Baihui (GV20), Fengfu (GV16)	Chinese Medicine theory - Previous studies	Right MCAO for 30 min	SD rats	1 day after MCAO; **Freq:** 5-25Hz; **Int:** 2.7-3mA; **Time:** 25min; **Duration:** once daily for 7 consecutive days	Acute	7	**1.** EA reduced infarct volume, **2.** EA reduced neurological deficit scores, **3.** EA downregulated GFAP expression, **4.** EA activated p38 MAPK/CREB pathway, **5.** EA upregulated expression of Bcl-xL and Bcl-1, **6.** EA downregulated expression of Bax, **7.** EA decreased Smac/DIABLO translocation, **8.** EA decreased caspase-3 upregulation, **9.** EA enhanced XIAP expression (all the results were shown at	EA downregulates reactive astrocytosis to provide neuroprotection by activating the p38 MAPK/CREB pathway. The modulating effects of EA on Bax-mediated apoptosis are possibly due to the activation of Bcl-xL and Bcl-2 signaling, thereby preventing Smac/DIABLO translocation and restoring XIAP-mediated caspase-3 inhibition

												the ischemic cortical penumbra)	
Electroacupuncture ameliorates cognitive impairment through inhibition of NF-kB mediated neuronal cell apoptosis in cerebral ischemia-reperfusion injured rats	Feng, X	Molecular Medicine Reports 7: 1516-1522, 2013	NF-kB pathway (-)	EA	Baihui (GV20), Shenting (GV24)	Chinese Medicine theory - Clinical use	Left MCAO for 120 min	SD rats	2 h after MCAO; **Freq:** 1-20Hz; **Time:** 30min; **Duration:** daily for 10 days	Acute	10	**1.** EA improved neurological deficit scores and reduced cognitive impairment (learning and memory), **2.** EA reduced infarct volume, **3.** EA inhibited apoptosis by blocking NF-κB pathway and downregulating the apoptotic Fas/Bax genes expression in ischemic tissue	EA inhibits NF-κB-mediated neuronal cell apoptosis

Electroacupuncture regulates TRPM7 expression through the trkA/PI3K pathway after cerebral ischemia–reperfusion in rats	Zhao, L	Life Sciences 81 (2007) 1211–1222	TRPM7 (-)	EA	Renzhong (GV26), Chengjiang (CV24)	Xingnao Kaiqiao acupuncture method	Right MCAO for 30 min	SD rats	Immediately after MCAO; **Freq:** 16-4Hz; **Int:** 1-3V; **Time:** 30min; **Duration:** single	Acute	1	**1.** EA reversed TRPM7 over-expression in ischemic cortex and hippocampal CA3 and CA1 regions, **2.** EA upregulated the expression of trkA in the same regions, **3.** trkA triggered the downstream PI3K pathway, necessary for the effect of EA on TRPM7	EA can reverse the increase of TRPM7 by enhancing trkA activity, which triggers the downstream PI3K pathway
Electroacupuncture effect on neurological behavior and tyrosine kinase-JAK 2 in rats with focal cerebral ischemia	Liu, R	Journal of Traditional Chinese Medicine 2012 September 15; 32(3): 1-2	JAK2 (-)	EA	Baihui (GV20), Dazhui (GV14)	Not specified	MCAO	SD rats	**Freq:** 20Hz; **Int:** 1-2mA; **Time:** 30min	Not specifi ed	1	**1.** EA improved neurological deficits, **2.** EA lowered the overexpression of JAK2 in the ischemic cortex	EA effectively inhibits the overexpression of JAK2, blocking the signal transduction pathways induced by JAK2 (neuronal apoptosis)
Effect of electroacupuncture on cell apoptosis and ERK signal pathway in the hippocampus of adult rats with cerebral ischemia-reperfusion	Wu, C	Evidence-Based Complementary and Alternative Medicine, Volume 2015, Article ID 414965	ERK (+)	EA	Chize (LU5)+Hegu (LI4); Zusanli (ST36)+ Sanyinjiao (SP6)	Clinical use	Left MCAO for 30 min	SD rats	1.5 h after MCAO; **Freq:** 2-15Hz; **Int:** 2mA; **Time:** 20min; **Duration:** once daily for 3 consecutive days	Acute	3	**1.** EA improved neurological deficits, **2.** EA diminished cerebral I/R injury, particularly in the CA1 hippocampus area, **3.** EA reduced the apoptosis index, and **4.** EA upregulated expression of p-ERK signaling pathway	EA alleviates neurological deficits, reduces the apoptosis index, and simultaneously upregulates the expression of p-ERK signaling pathway in I/R rats

Category IV: Regulation of neurochemicals

Table S7: Neurotransmitters and receptors

Study title	Authors	Publication	Target (receptor/molecule/ pathway)	Ac type	Acupoints	Selection reason	Experimental model	Animal	Treatment	Stage	# sessions	Results	Conclusions
Acupuncture stimulation at Baihui acupoint reduced cerebral infarct and increased dopamine levels in chronic cerebral hypoperfusion and ischemia-reperfusion injured Sprague-Dawley rats	Chuang, C	The American Journal of Chinese Medicine, Vol. 35, No. 5, 779–791, 2007	Dopamine (+)	MA	Baihui (GV20)	Chinese Medicine theory - Previous studies	Right MCAO for 90 min	SD rats	Immediately after MCAO; **Time:** 20min; **Duration:** 3 times a week for 4 weeks	Acute	12	**1.** Ac did not improve cognitive and memory deficits in rats with ischemia reperfusion injury, **2.** Ac increased dopamine levels in the right cerebral cortex and hippocampus	Ac increases levels of dopamine reducing the degree of cerebral atrophy after cerebral infarct
Role of GABA in electro-acupuncture therapy on cerebral ischemia induced by occlusion of the middle cerebral artery in rats	Gan, P	Neuroscience Letters 383 (2005) 317–321	GABA (+)	EA	Shuigou (GV26), Baihui (GV20)	Not specified	Left MCAO for 120 min	SD rats	15 min after MCAO; **Freq:** 3.58-6.25Hz; **Int:** 1.4-2mA; **Time:** 60min with a 10 min pause every 30 min; **Duration:** single	Acute	1	**1.** EA increased GABA in cerebral cortex and hippocampus CA1 region, **2.** EA reduced infarct size, **3.** EA increased the percentage of surviving neurons in the ipsilateral cerebral cortex and striatum	EA up-regulates GABA expression in the ischemic cerebral cortex and CA1 hippocampus area

Acupuncture improves locomotor function by enhancing GABA receptor expression in transient focal cerebral ischemia rats	Xu, Q	Neuroscience Letters 588 (2015) 88–94	GABA receptors (+)	MA	Jiaji (EX-B2)	Previous studies	Right MCAO	SD rats	**Stimulation:** Rotated clockwise 2-3 times per sec; **Time:** 30sec; **Duration:** 7 days	Not specified	7	**1.** Ac improved performance in behavioral tests, **2.** AC reduced infarct volume, **3.** AC increased β-endorphin concentrations in striatum and spinal cord, and **4.** Ac increased expression of GABA receptors in striatum and spinal cord	AC improves locomotor function by modulating the expression of GABA receptors
Electroacupuncture Attenuates Both Glutamate Release and Hyperemia After Transient Ischemia in Gerbils	Pang, J	The American Journal of Chinese Medicine, Vol. 31, No. 2, 295–303, 2003	Glutamate (-)	EA	Fengfu (GV16), Shendao (GV11)	Not specified	Bilateral common carotid artery occlusion for 5 min	Gerbils	Immediately after ischemia; **Freq:** 7Hz; **Int:** 6mA; **Time:** 30min	Acute	1	**1.** EA depressed glutamate release in CA1 hippocampal subfield, **2.** EA suppressed hyperemia during reperfusion	EA suppresses both hyperemia and excessive glutamate release during and after ischemia
Electroacupuncture regulates NMDA receptor NR1 subunit expression via PI3-K pathway in a rat model of cerebral ischemia–reperfusion	Sun, N	Brain Research 1064 (2005) 98–107	NMDA receptor (-)	EA	Renzhong (GV26), Chengjiang (CV24)	Not specified	Right MCAO for 30 min	SD rats	Immediately after MCAO; **Freq:** 16-4Hz; **Int:** 1-3V; **Time:** 30min; **Duration:** single	Acute	1	**1.** EA reduced NR1 levels in ischemic cortical areas through PI3K, **2.** EA increased TrkA in cortical ischemic areas	EA prolongs the time windows of the expression of TrkA in ischemic cortex. EA also reverses the expression of the NR1 subunit through the PI3-K pathway

Table S8: Antioxidant enzymes

Study title	Authors	Publication	Target (receptor/ molecule/ pathway)	Ac type	Acupoints	Selection reason	Experimental model	Animal	Treatment	Stage	# sessions	Results	Conclusions
Acupuncture prevents cognitive deficits and oxidative stress in cerebral multi-infarction rats	Liu, C	Neuroscience Letters 393 (2006) 45–50	SOD/ GSH-Px (+)	MA	Tanzhong (REN17), Zhongwan (REN12), Qihai (REN6), Zusanli (ST36), Xuehai (SP10)	Yiqitiaoxue-Fubenpeiyuan acupuncture method	Internal carotid occlusion over 1-2 min	Wistar rats	9 days after occlusion; **Stimulation:** twisted twice a sec. for 30 sec. in each point; **Duration:** Once daily for 21 days (rest every 7 days)	Sub-acute	18	**1.** Ac significantly increased hippocampal SOD and GSH-Px activity	Ac exerts beneficial effects on spatial memory and antioxidant status of cerebral multi-infarction rats

Acupuncture elicits neuroprotective effect by inhibiting NAPDH oxidase-mediated reactive oxygen species production in cerebral ischemia	Shi, G	Scientific Reports, December 2015, 5:17981, DOI: 10.1038	NADPH oxidase (-)	MA	Bilateral Zusanli (ST36), Baihui (GV20)	Not specified	Common carotid artery occlusion	Wistar rats	3 days after occlusion; **Stimulation:** Twirling manipulation >60 in 30 sec; **Duration:** once daily for 2 weeks (1 day rest after 6 days of treatment)	Sub-acute	13	**1.** Ac ameliorated neurological impairment, **2.** Ac reduced infarct size, **3.** Ac suppressed overproduction of O_2 via inhibition of NADPH oxidase in the hippocampus, and **4.** Ac suppressed expression of NADPH oxidase subunits	Ac plays a neuro-protective effect against cognitive impairment via inhibition of NAPDH oxidase-mediated oxidative stress
Electroacupuncture reduces the extent of lipid peroxidation by increasing superoxide dismutase and glutathione peroxidase activities in ischemic reperfused rat brains	Siu, F	Neuroscience Letters 354 (2004) 158–162	Antioxidant enzymes (+)	EA	Fengchi (GB20) bilateral	Not specified	Right MCAO for 1h	SD rats	30 min after MCAO; **Freq:** 2Hz; **Int:** 0.7V; **Time:** 30min; **Duration:** single	Acute	1	**1.** EA increased SOD and GSH-Px activity in ischemic brain	EA reduces the extent of lipid peroxidation in ischemic-reperfused rat brains, possibly by increasing the activities of SOD and GPx
Neurochemical mechanism of electroacupuncture: anti-injury effect on cerebral function after focal cerebral ischemia in rats	Zhong, S	Advance Access Publication 27 October 2007 eCAM 2009; 6(1)51–56	Respiratory chain enzymes	EA	Renzhong (GV26), Baihui (GV20)	Not specified	Right MCAO for 90 min	SD rats	Immediately after MCAO; **Freq:** 5/20Hz; **Int:** 2-4mA; **Time:** 60min with 15 min rest every 20 min; **Duration:** single	Acute	1	**1.** EA reduced neurological deficits, **2.** improved mitochondrial respiratory control ratio in penumbra area, and **3.** EA increased the activities of succinic dehydrogenas	EA improves respiratory chain function and has an anti-oxidative action in brain tissues at the infarct penumbra zone

												e, NADH dehydrogenase and cytochrome C oxidase in penumbra zone	
Electroacupuncture potentiates the disulphide-reducing activities of thioredoxin system by increasing thioredoxin expression in ischemia-reperfused rat brains	Siu, F	Life Sciences 77 (2005) 386–399	Thioredoxin (+)	EA	Fengchi (GB20), bilateral Zusanli (ST36)	Clinical use	Right MCAO for 1h	SD rats	30 min after MCAO; **Freq:** 2Hz; **Int:** 0.7V; **Time:** 30min; **Duration:** single	Acute	1	**1.** EA increased TR activity with St36, **2.** EA upregulated expression of Trx at both acupoints	EA can upregulate the activity of Trx system by increasing the availability of Trx to TR and thereby reduce the ROS-induced formation of disulphides

Table S9: Inflammatory mediators

Study title	Authors	Publication	Target (receptor/ molecule/ pathway)	Ac type	Acupoints	Selection reason	Experimental model	Animal	Treatment	Stage	# sessions	Results	Conclusions
Effects of acupuncture at Baihui (DU20) and Zusanli (ST36) on the expression of heat shock protein 70 and tumor necrosis factor α in the peripheral serum of cerebral ischemia-	Xu, H	Chinese Journal of Integrative Medicine 2014 May;20(5):369-374	HSP70/ TNFα (-)	EA	Left Zusanli (ST36), Baihui (GV20)	Chinese Medicine theory - Clinical use	Right MCAO for 2h	SD rats	After 2 h MCAO; **Freq:** 2Hz; **Int:** 1mA; **Time:** 20min; **Duration:** once a day (7 times in total)	Acute	7	**1.** Ac reduced the expression of HSP70 and TNF-α protein in the peripheral serum	Ac delays inflammatory injury, which was associated with a reduction in the expression of HSP70 and TNF-α

reperfusion-injured rats													
Electroacupuncture exerts anti-inflammatory effects in cerebral ischemia reperfusion injured rats via suppression of the TLR4/NF-kB pathway	Lan, L	International Journal of Molecular Medicine 31: 75-80, 2013	TLR4/ NF-kB (-)	EA	Zusanli (ST36), Quchi (LI11) on the paretic limb	Clinical use	Left MCAO for 120 min	SD rats	2 h after MCAO; **Freq:** 1-20Hz; **Int:** 0.01mA; **Duration:** 1 day	Acute	1	**1.** EA improved neurological function, **2.** reduced infarct volume, **3.** alleviated cerebral inflammation, **4.** suppressed activation of the TLR4/NF-kB pathway by inhibiting NF-kB nuclear translocation, and **5.** EA inhibited I/R-induced secretion of TNFα, IL-1β, IL-6	EA reduces ischemic brain damage, improves neurological deficits and exerts anti-inflammation activity via inhibition of the TLR4/NF-κB pathway

Table S10: Neurotrophic factors

Study title	Authors	Publication	Target (receptor/ molecule/pathway)	Ac type	Acupoints	Selection reason	Experimental model	Animal	Treatment	Stage	# sessions	Results	Conclusions

Electroacupuncture enhances motor recovery performance with brain-derived neurotrophic factor expression in rats with cerebral infarction	Kim, M	Acupuncture in Medicine Journal 2012;30:222–226	BDNF (+)	EA	Baihui (GV20), on the non paretic side Qubin (GB7)	Not specified	Right MCAO for 2h	SD rats	**Freq:** 3Hz; **Time:** 5min; **Duration:** every 2 days for 2 weeks	Not specified	6	**1.** EA improved motor recovery, **2.** enhanced expression of BDNF in the ischemic lobe, and **3.** EA increased TrkB	EA increases the expression of BDNF and improves motor recovery

Table S11: Anaerobic metabolism

Study title	Authors	Publication	Target (receptor/ molecule/ pathway)	Ac type	Acupoints	Selection reason	Experimental model	Animal	Treatment	Stage	# sessions	Results	Conclusions

Electroacupuncture up-regulates astrocytic MCT1 expression to improve neurological deficit in middle cerebral artery occlusion rats	Lu, Y	Life Sciences 134 (2015) 68–72	Lactate (+)	EA	Bilateral Neiguan (PC6), bilateral Quchi (LI11)	Not specified	MCAO	Wistar rats	After 2h of MCAO; **Freq:** 2/15Hz; **Int:** 1mA; **Time:** 20min; **Duration:** once daily for 7 days	Acute	7	**1.** EA promoted recovery of neurological deficits, **2.** increased lactate concentrations in the ischemic brain, **3.** enhanced expression of MCT1 in astrocytes, and **4.** EA increased GFAP expression in the ischemic area, mainly at the hippocampus	EA improves neurological deficits through activating the lactate metabolism in the ischemic brain area and up-regulating the astrocytic expression of MCT1 to facilitate the transfer of intracellular lactate to the extracellular domain to be used by injured neurons

Category V: Memory improvement

Table S12: LTP modulation

Study title	Authors	Publication	Target (receptor/ molecule/ pathway)	Ac type	Acupoints	Selection reason	Experimental model	Animal	Treatment	Stage	# sessions	Results	Conclusions
Hippocampal cAMP/PKA/CREB is required for neuroprotective effect of acupuncture	Li, Q	Physiology & Behavior 139 (2015) 482–490	cAMP/ PKA/ CREB (+)	MA	Zusanli (ST36) bilateral	Previous studies	Right internal carotid occlusion	Wistar rats	3 days after occlusion; **Stimulation:** Turned two spins per second for 30 seconds; **Duration:** once daily for 14 days (rest every 7 days)	Sub-acute	13	**1.** Ac improved hippocampal-dependent memory, **2.** Ac. reversed impairment of LTP in hippocampal PP-DG by upregulating cAMP/PKA/CREB pathway, **3.** Ac. significantly inhibited PDE activity, and **4.** Ac. enhanced ERK expression in CA1 and DG regions	Acupuncture improves cognitive hippocampus function by modulating the cAMP/PKA/CREB signaling pathway
Electroacupuncture at Baihui acupoint (GV20) reverses behavior deficit and long term potentiation through N-Methyl-D-Aspartate and transient receptor potential vanilloid subtype 1 receptors in middle cerebral artery occlusion rats	Lin, Y	Journal of Integrative Neuroscience. Volume 9, Number 3, September 2010, 269-282	NR1-TRPV1 (-)	EA	Baihui (GV20)	Previous studies	Right MCAO for 10 min	SD rats	1 day after MCAO; **Freq:** 2Hz; **Int:** 2mA; **Time:** 20min; **Duration:** 6 days	Acute	6	**1.** EA improved behavior deficit, **2.** EA restored LTP in hippocampal CA1 areas, **3.** EA decreased NR1 and TRPV1 levels in hippocampal CA1 areas (pyramidal neurons) to basal conditions	EA improves vascular dementia by decreasing the expression of NR1-TRPV1
